# Determinants of study completion and response to a 12-month behavioral physical activity intervention in chronic obstructive pulmonary disease: A cohort study

**DOI:** 10.1371/journal.pone.0217157

**Published:** 2019-05-20

**Authors:** Maria Koreny, Heleen Demeyer, Ane Arbillaga-Etxarri, Elena Gimeno-Santos, Anael Barberan-Garcia, Marta Benet, Eva Balcells, Eulàlia Borrell, Alicia Marin, Diego A. Rodríguez Chiaradía, Pere Vall-Casas, Jordi Vilaró, Robert Rodríguez-Roisin, Judith Garcia-Aymerich

**Affiliations:** 1 ISGlobal, Barcelona, Spain; 2 Pompeu Fabra University (UPF), Barcelona, Spain; 3 CIBER Epidemiología y Salud Pública (CIBERESP), Barcelona, Spain; 4 Department of Rehabilitation Sciences, KU Leuven -University of Leuven, Leuven, Belgium; 5 Department of Respiratory Diseases, University Hospitals KU Leuven, Leuven, Belgium; 6 Physical Activity and Sports Sciences, Faculty of Psychology and Education, University of Deusto, Donostia-San Sebastián, Spain; 7 Respiratory Clinic Institute, Hospital Clinic of Barcelona, Barcelona, Spain; 8 August Pi i Sunyer Biomedical Research Institute (IDIBAPS), Barcelona, Spain; 9 CIBER Enfermedades Respiratorias (CIBERES), Madrid, Spain; 10 Pneumology Department, Institut Hospital del Mar d'Investigacions Mèdiques (IMIM), Hospital del Mar, Barcelona, Spain; 11 Sant Roc Primary Health Care Centre, Institut Català de la Salut (ICS), Badalona, Spain; 12 Department of Pulmonary Medicine, Hospital Universitari Germans Trias i Pujol, Badalona, Spain; 13 Fundació Institut d’Investigació en Ciències de la Salut Germans Trias i Pujol, Badalona, Spain; 14 Universitat Internacional de Catalunya (UIC), Barcelona, Spain; 15 Global Research on Wellbeing (GRoW). Blanquerna Health Sciences School, Ramon Llull University, Barcelona, Spain; Srebrnjak Children's Hospital, CROATIA

## Abstract

**Objectives:**

Physical activity is key to improve the prognosis of chronic obstructive pulmonary disease (COPD). To help to tailor future interventions we aimed to identify the baseline characteristics of COPD patients which predict 12-month completion and response to a behavioral physical activity intervention.

**Methods:**

This is a 12-month cohort study of the intervention arm of the Urban Training randomized controlled trial (NCT01897298), an intervention proven to be efficacious to increase physical activity. We considered baseline sociodemographic, interpersonal, environmental, clinical and psychological characteristics as potential determinants of completion and response. We defined completion as attending the 12-month study visit. Among completers, we defined response as increasing physical activity ≥1100 steps/day from baseline to 12 months, measured by accelerometer. We estimated the factors independently for completion and response using multivariable logistic regression models.

**Results:**

Of a total of 202 patients (m (SD) 69 (9) years, 84% male), 132 (65%) completed the study. Among those, 37 (28%) qualified as responders. Higher numbers of baseline steps/day (OR [95% CI] 1.11 [1.02–1.21] per increase of 1000 steps, p<0.05) and living with a partner (2.77 [1.41–5.48], p<0.01) were related to a higher probability of completion while more neighborhood vulnerability (0.70 [0.57–0.86] per increase of 0.1 units in urban vulnerability index, p<0.01) was related to a lower probability. Among the completers, working (3.14 [1.05–9.33], p<0.05) and having an endocrino-metabolic disease (4.36 [1.49–12.80], p<0.01) were related to a higher probability of response while unwillingness to follow the intervention (0.21 [0.05–0.98], p<0.05) was related to a lower probability.

**Conclusions:**

This study found that 12-month completion of a behavioral physical activity intervention was generally determined by previous physical activity habits as well as interpersonal and environmental physical activity facilitators while response was related to diverse factors thought to modify the individual motivation to change to an active lifestyle.

## Introduction

Chronic obstructive pulmonary disease (COPD) is a leading cause of morbidity and mortality worldwide [1]. Over the last decades, physical activity has been increasingly recognized as a key factor for COPD for two main reasons. First, higher levels of physical activity practice in daily life have been consistently related to improved prognosis in COPD, specifically to reductions in exacerbations and mortality [2]. Second, in comparison with healthy peers, COPD patients exhibit reduced levels of physical activity already in the early stages [3] and across all other airflow limitation severity stages [4]. As a result, the guidelines for the prevention and management of COPD developed by the Global Initiative for Chronic Obstructive Lung Disease (GOLD) 2017 include physical activity as a non-pharmacological therapeutic approach that all COPD patients should receive [5].

Recent efforts have succeeded in designing and testing interventions that increased physical activity in COPD patients either at short [6–8] or long-term [9]. However, research is still scarce and there are no clear guidelines on how, when, where or to whom such interventions should be provided [10]. A critical issue is to ensure that a relevant proportion of patients completes the intervention period and responds to the intervention (e.g. with an increase in the targeted outcome above a critical threshold). Unfortunately, research of this kind in physical activity and COPD is scarce. To our knowledge, four studies have assessed factors that affect completion and/or response to a physical activity intervention in COPD in *post hoc* or secondary analyses. Altenburg et al showed that only patients with baseline physical activity <10,000 steps/day maintained a significant long-term effect at 15 months of a 12-week physical activity counselling program [11]. Moy et al found that, among a large number of clinical factors, only age was associated with changes in steps/day at 4 months of an internet-mediated, pedometer-based exercise intervention [12]. Demeyer et al reported that the magnitude of the response to a 12-week semiautomated telecoaching program was higher in patients who exhibited at baseline lower dyspnea scores, better exercise capacity and who suffered from mild-to-moderate COPD [8]. This research also found that study completion was associated with higher body mass index (BMI), higher isometric quadriceps force and a better health status [8]. Finally, Kantorowski et al recently found that lower baseline physical activity, social support, lack of depression or oxygen use, and recruitment in spring were significant predictors of a positive change (≥1 step/day) in physical activity after a 12-week pedometer and website intervention (vs pedometer only) [13]. Overall, these studies seem to suggest that lower baseline physical activity and better functional performance relate to a better response and completion.

An important characteristic of the mentioned trials is that they were limited to COPD patients with reduced functional exercise capacity (median 6-min walking distance (6MWD) test ≈ 450m [8,11] or below [13]) and/or low baseline physical activity [8,11–13]. Therefore, their results on completion and response to physical activity interventions may not be transferable to other patients. Another shortcoming in previous studies is that they mostly focused on characteristic features of COPD to predict completion and/or response. These characteristics do not reflect the complex interplay of factors affecting physical activity as described by the ecological framework [14].

According to the World Health Organization (WHO) strategy for chronic medical conditions, the outcomes of health promotion and secondary prevention interventions strongly depend on the involvement of patients and their families [15]. Thus, the deployment at the population level of interventions originally assessed at clinical trial level requires a proper assessment of the determinants of completion and response using a broad approach and involving patients across all severity levels as typically found in the community setting.

The aim of our study was to identify which sociodemographic, interpersonal, environmental, clinical, and psychological baseline characteristics were associated with 12-month completion and response to a behavioral physical activity intervention in COPD patients, using data from the intervention arm of the randomized controlled Urban Training trial (NCT01897298) mostly involving COPD patients from primary care. This intervention has previously been reported to be efficacious to increase physical activity in the per protocol population but ineffective in the full population [9], which prompts the need to identify the profile of future completers and/or responders. Ideally, the present results will help to improve clinical guidance on how to tailor behavioral physical activity interventions in COPD patients.

## Methods

### Design and subjects

We performed a cohort study of 12-month follow-up of the intervention group participating in the Urban Training trial behavioral physical activity intervention in COPD patients. This was a prospective, multicenter, parallel-group, randomized controlled trial, registered at clinicaltrials.gov (NCT01897298) and previously described in detail [9]. In brief, the Urban Training trial recruited 407 patients with a diagnosis of COPD according to the American Thoracic Society and European Respiratory Society (ATS/ERS) recommendations (post- bronchodilator forced expiratory volume in the first second (FEV_1_) to forced vital capacity (FVC) ratio <0.70) [16] from 33 primary care and 5 tertiary hospitals of five Catalan seaside municipalities between October 2013 and January 2016 and randomized them to the intervention or usual care group. For the present study we included only patients in the intervention arm (n = 202).

The Urban Training trial was approved by the Ethics Committees of all participating institutions (Comitè Ètic d‘Investigació Clínica Parc de Salut MAR 2011/4291/I, Comitè Ètic d‘Investigació Clínica de l’IDIAP Jordi Gol i Gurina P11/116, Comitè Ètic d‘Investigació Clínica de l’Hospital Universitari de Bellvitge PR197/11, Comitè Ètic d‘Investigació Clínica de l’Hospital Universitari Germans Trias i Pujol AC-12-004, Comitè Ètic d‘Investigació Clínica de l’Hospital Clínic de Barcelona 2011/7061, Comitè Ètic d‘Investigació Clínica de l’Hospital de Mataró November 23^rd^, 2011) and all participants provided written informed consent.

### Intervention

The Urban Training intervention included six elements aiming to elicit a behavioral change and to maintain it during the 12 months of the study: (1) a motivational interview [17], following a stage-matched approach [18], with up to four short motivational phone calls during the subsequent 12 months; (2) instructions to walk at least one of the previously validated [19] Urban Training walking trails per day, at least 5 days per week on the appropriate intensity trail with tailored increase of volume and/or intensity over 12 months; (3) a pedometer and a personalised calendar to monitor and note their physical activity and keep motivation; (4) the project website [20] and phone text messages every 2 weeks with educational or motivational messages and the European Lung Foundation information brochure [21]; (5) a guided walking group once per month; and (6) a support phone number available during the 12-month study period.

### Procedures

Full details on study procedures and quality control have been reported previously [9,22]. Briefly, we obtained at baseline the following data from all patients using standardized procedures: (i) sociodemographic variables: age, sex, smoking and socioeconomic status; (ii) interpersonal variables: living with a partner (vs single, widowed or divorced), grandparenting and working status (working full-time or part-time vs unemployed, housework or retired); (iii) environmental variables: urban vulnerability index, a measure of socioeconomic status at the census tract level (median area of 0.26 km^2^) that combines demographic, economic, residential and subjective indicators, and ranges from 0 (lowest) to 1 (highest level of neighborhood vulnerability) [23], and season of recruitment; (iv) clinical variables: forced expiratory volume in 1 second (FEV_1_) and forced vital capacity (FVC) by forced spirometry after bronchodilator, functional exercise capacity measured by the 6-min walking distance (6MWD) test, the modified Medical Research Council dyspnea scale (mMRC), the number of severe COPD exacerbations (defined as requiring a visit to the emergency room or hospital admission) in the 12 months prior to recruitment and during follow-up, body mass index (BMI) and fat free mass index (FFMI) by physical examination and bioelectrical impedance, physician diagnosed comorbidities categorized according to the International Classification of Diseases, Tenth Revision ICD-10 (C00 to D48 for Neoplasm; E00 to E90 for Endocrine, nutritional and metabolic diseases; E10 to E14 for Diabetes mellitus; I00 to I99 for Cardiovascular diseases; I10 to I15 for Hypertension) and physical activity variables: steps/day and time spent on moderate to vigorous physical activity (>3 METs [metabolic equivalents of task]s) measured by the Dynaport accelerometer (McRoberts BV, The Hague, The Netherlands) previously validated for COPD [24,25], defining a valid physical activity measurement as a minimum of 3 days with at least 8 h of wearing time within waking hours [26], and physical activity experience (total and amount and difficulty domains) by the Clinical-PROactive Physical Activity (C-PPAC) tool [27]; and (v) psychological variables: the Hospital Anxiety and Depression scale (HADs), unwillingness to follow the physical activity intervention (i.e. patients who ‘*spontaneously reported at baseline that they were unwilling to follow any of the instructions’* [9]), stage of change [17,18] and self-efficacy (from 0 to 10).

### Study outcomes

We defined study completion as participation in the final visit (12 months after the baseline visit). Among patients who completed the study, we defined intervention response as a positive change in mean daily step count between baseline (visit 2) and 12 months (visit 4) equal or higher than the minimal important difference (MID) [28]. Steps MID in COPD has been estimated to range between 600 and 1100 steps/day [28]; therefore we used a cut-off of ≥1100 vs <1100 steps/day for the main analysis and ≥600 vs <600 steps/day for the secondary analysis.

### Statistical analysis

The available sample size (n = 202) was fixed by the primary objectives of the Urban Training study, therefore we calculated the statistical power to answer the current research question. Calculations were performed for a difference in age, FEV_1_, FVC, 6MWD, and steps/day at baseline and resulted in a range of statistical power between 93% to 98% for study completion and 79% to 90% for response, using unpaired t-test (p<0.05) and assuming a relation of loss-to follow-up to completion and responders to non-responders of 1:2.

We assessed the presence and patterns of missing data. Due to the small proportion of missings (<5% of total data), main analysis was conducted using a complete case strategy and we reported missing data in the Table footnotes. As a sensitivity analysis, to account for the possibility of bias due to missing data, we used multiple imputation (20 imputed datasets) with the method of chained equations [29] assuming the missing-at-random hypothesis (i.e., missingness conditional on measured patients’ characteristics [30]). We repeated bivariable and multivariable analysis using the imputed datasets.

To analyze determinants of study completion and response to intervention, we first compared the individual’s characteristics (sociodemographic, interpersonal, environmental, clinical and psychological) at baseline between patients lost to follow-up and completers, and between non-responders and responders using unpaired Student’s t-test, Wilcoxon rank-sum, Chi square or Fisher’s tests, depending on the variable distribution. Second, we built two multivariable logistic regression models (one for study completion and one for response to the intervention) including as exposures all variables that exhibited a p-value <0.2 in the bivariable analysis between lost to follow-up and completers, and non-responders and responders, respectively. Model building combined step-forward and backward algorithms, and covariates were included in the final model if: (i) they related to both the exposure and the outcome in bivariable analysis; (ii) they modified (>10% change in regression coefficient) the estimates of the remaining variables in the multivariable models; or, (iii) there was consistent evidence in the literature of their association with the outcome [31]. To exclude overfitting, we applied the least absolute shrinkage and selection operator (LASSO) approach. We tested goodness of fit of the final models by means of the Hosmer-Lemeshow test, identification of influential observations, and estimation of the specification error. Finally, we tested the discrimination and accuracy to predict the outcome of the final models by calculating the area under the receiver operating characteristic curve (AUROC) and the Brier score, respectively.

We conducted several secondary analyses. First, we used 600 steps (lower limit of steps MID) as a cut-off to define response to the intervention both in bivariable and multivariable analyses. Second, we repeated the final multivariable model for response: (1) including severe acute exacerbations of COPD during follow-up in the multivariable models to test the possibility that this variable affected the risk of being a non-responder; and (2) using time in moderate to vigorous physical activity (categorized according to its median value) instead of steps to define response to the intervention.

All analyses were conducted with Stata 12.0 (StataCorp, College Station, TX, USA).

## Results

### Patient characteristics

Patient characteristics are shown in Table 1. Patients had a mean (SD) age of 69 (9) years and were mostly male (84%) and living with a partner (72%). Only 14% were active workers and 71% had low socioeconomic status. Patients were mainly in the earlier stages of COPD with a mean FEV_1_ 56% of predicted and a mean 6MWD of 487m; 29% of patients had an mMRC dyspnea score ≥2, 9% of patients had a severe COPD exacerbation within the previous year and only 6% were participating in a pulmonary rehabilitation program at study inclusion. Patients had a large prevalence of endocrino-metabolic and cardiovascular chronic comorbid conditions and walked more than 7000 steps per day on average. At baseline, 26 patients (13%) reported unwillingness to follow the intervention, 50% of patients were in later phases of stage of change (action, maintaining and finalizing), and self-efficacy was high (median 8 on a scale from 0 to 10).

**Table 1:** Variables related to 12-month completion in COPD patients participating in a behavioral physical activity intervention.

	All patients	Lost to follow-up	Completers	p-value
	n = 202*	n = 70	n = 132	
**Sociodemographic**				
Age (years), m (SD)	68.8 (9.2)	69.9 (9.3)	68.3 (9.1)	0.229
Sex: male, n (%)	170 (84)	56 (80)	114 (86)	0.239
Smoking status, current, n (%)	56 (28)	22 (31)	34 (26)	0.392
Socioeconomic status, IIIM-IV-V, n (%)	143 (71)	50 (71)	93 (71)	0.948
**Interpersonal**				
Living with a partner**, n (%)	145 (72)	41 (59)	104 (79)	0.004
Grandparenting, n (%)	68 (34)	21 (31)	47 (36)	0.481
Active workers, n (%)	28 (14)	9 (13)	19 (14)	0.764
**Environmental**				
Urban vulnerability index (from 0 -lowest to 1 –highest), m (SD)	0.64 (0.17)	0.69 (0.16)	0.61 (0.17)	0.003
Recruitment season				
Spring, n (%)	46 (23)	13 (19)	33 (25)	0.305
Summer, n (%)	22 (11)	11 (16)	11 (8)	
Fall, n (%)	73 (36)	27 (38)	46 (35)	
Winter, n (%)	61 (30)	19 (27)	42 (32)	
**Clinical**				
FEV_1_ (% pred), m (SD)	56.4 (17.1)	57.2 (17.9)	55.9 (16.7)	0.616
FVC (% pred), m (SD)	77.3 (16.8)	76.9 (16.8)	77.5 (16.9)	0.817
6MWD (m), m (SD)	487 (98)	464 (102)	498 (95)	0.018
Moderate to very severe dyspnea (mMRC ≥2), n (%)	58 (29)	22 (31)	36 (27)	0.534
Any severe^¶^ COPD exacerbation in previous 12 months, n (%)	17 (9)	7 (11)	10 (8)	0.591
Any severe^¶^ COPD exacerbation during follow-up, n (%)	22 (17)	-	22 (17)	-
BMI (kg/m^2^), m (SD)	28.5 (5.0)	28.6 (5.1)	28.4 (4.9)	0.812
FFMI, m (SD)	19.6 (3.2)	19.6 (3.5)	19.6 (3.0)	0.978
Neoplasm^†^, n (%)	24 (12)	5 (8)	19 (15)	0.248
Endocrine, nutritional and metabolic diseases^†^, n (%)	131 (67)	42 (64)	89 (68)	0.546
Diabetes mellitus^†^, n (%)	61 (31)	17 (26)	44 (34)	0.262
Cardiovascular disease^†^, n (%)	124 (63)	43 (65)	81 (62)	0.649
Hypertension^†^, n (%)	94 (48)	33 (50)	61 (47)	0.649
Steps/day, m (SD)	7488 (4234)	6395 (3315)	8069 (4554)	0.007
Time in moderate to vigorous physical activity (>3 METs; h/day), med (P25-P75)	1.7 (1.2–2.2)	1.6 (1.1–2.0)	1.7 (1.3–2.4)	0.058
Intensity during physical activities (m/s^2^), m (SD)	1.029 (0.265)	0.970 (0.229)	1.060 (0.279)	0.020
C-PPAC amount, med (P25-P75)	77 (67–83)	72 (65–83)	77 (67–83)	0.223
C-PPAC difficulty, med (P25-P75)	83 (72–94)	81 (69–94)	86 (75–94)	0.562
C-PPAC score, med (P25-P75)	78 (70–86)	78 (67–84)	78 (72–86)	0.265
**Psychological**				
Anxiety (HAD-A), m (SD)	5.4 (4.2)	5.6 (4.6)	5.3 (4.0)	0.637
Depression (HAD-D), m (SD)	3.6 (3.7)	3.3 (4.1)	3.8 (3.4)	0.449
Unwillingness to follow the intervention, n (%)	26 (13)	2 (3)	24 (18)	0.002
Stage of change: action, maintaining, finalizing^§^, n (%)	80 (50)	22 (41)	58 (55)	0.095
Self-efficacy^‡^ (0 to 10), med (P25-P75)	8 (7–10)	9 (7–10)	8 (7–10)	0.920

Data are presented as n (%), mean (SD) or median (P25-P75). FEV_1_: forced expiratory volume in 1 second; FVC: forced vital capacity; 6MWD: 6-min walking distance; mMRC: modified Medical Research Council; BMI: body mass index; FFMI: fat free mass index; MET: metabolic equivalent of task; C-PPAC: Clinical visit—PROactive Physical Activity in COPD; HAD-A: Hospital Anxiety and Depression scale- Anxiety; HAD-D: Hospital Anxiety and Depression scale- Depression.

** Living with a partner vs single, widowed or divorced.

^¶^ A COPD exacerbation was considered severe if the patient required admission to the hospital or the emergency department.

^†^ ICD10 codes: C00 to D48 for Neoplasm; E00 to E90 for Endocrine, nutritional and metabolic diseases; E10 to E14 for Diabetes mellitus; I00 to I99 for Cardiovascular diseases; I10 to I15 for Hypertension.

^§^ Stage of change: action, maintaining, finalizing vs pre-contemplation, contemplation, preparation.

^‡^ Self-efficacy: Sure to go out for a walk every day (0 not sure- 10 completely sure).

* Some variables had missing values. Number of missings for 12-month completion: 1 in socioeconomic status, 1 in marital status, 3 in grandparenting, 3 in urban vulnerability index, 7 for severe COPD exacerbation in previous 12 months, 16 for FFMI, 5 for neoplasm, 5 for endocrine, nutritional and metabolic diseases, 5 for diabetes mellitus, 5 for any cardiovascular disease, 5 for hypertension, 57 for C-PPAC, 1 in depression, 42 in stage of change, 14 in self-efficacy.

### Determinants of study completion

**Fig 1:**
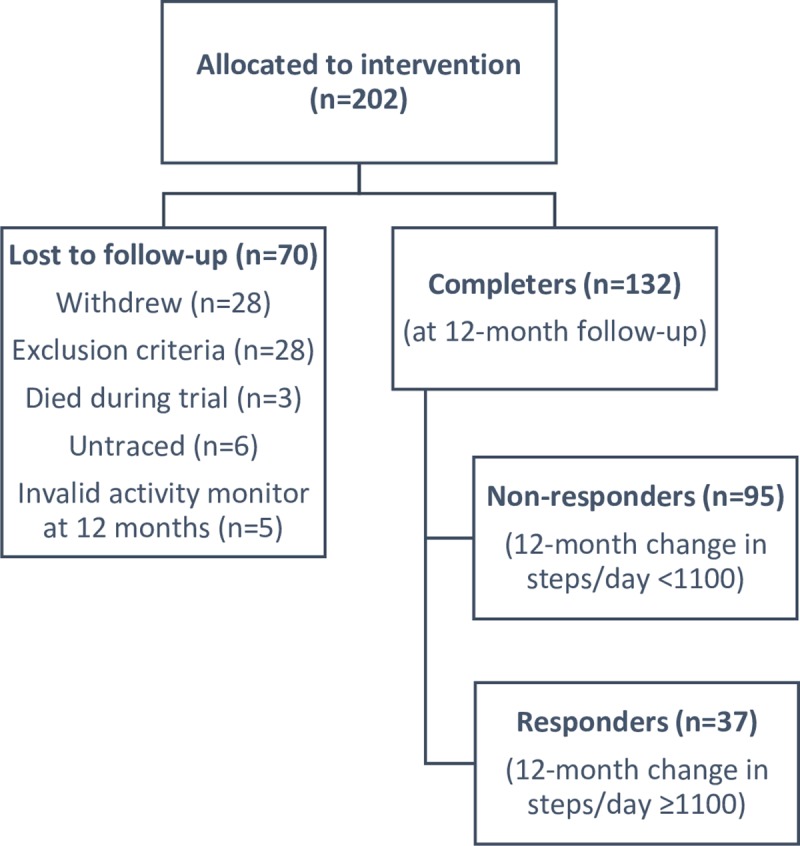
Flow of participants through the study.

After 12 months, 70 patients (35%) were lost to follow-up and 132 patients (65%) completed the study (completers) (Fig 1).

Of the broad range of variables assessed at baseline, physical activity levels, marital status, neighborhood vulnerability, unwillingness to follow the intervention and baseline 6MWD were significantly different between patients lost to follow-up and completers (Table 1). In the mutually adjusted logistic regression model, being physically more active (OR [95% CI] 1.11 [1.02–1.21] per increase of 1000 steps, p = 0.012) and living with a partner (2.77 [1.41–5.48], p = 0.003) were independently related to a higher probability of completion while more neighborhood vulnerability (0.70 [0.57–0.86] per increase of 0.1 units in urban vulnerability index, p = 0.001) was related to a lower probability (Table 2).

**Table 2:** Adjusted predictive factors of 12-month completion of a behavioral physical activity intervention in 202 COPD patients.

	OR (95% CI)	p-value
Steps/day (per increase of 1000 steps)	1.11 (1.02–1.21)	0.012
Living with a partner (vs single/ widowed/ divorced)	2.77 (1.41–5.48)	0.003
Urban vulnerability index (per increase of 0.1 units)	0.70 (0.57–0.86)	0.001

OR: odds ratio; CI: confidence interval.

### Determinants of intervention response

**Fig 2:**
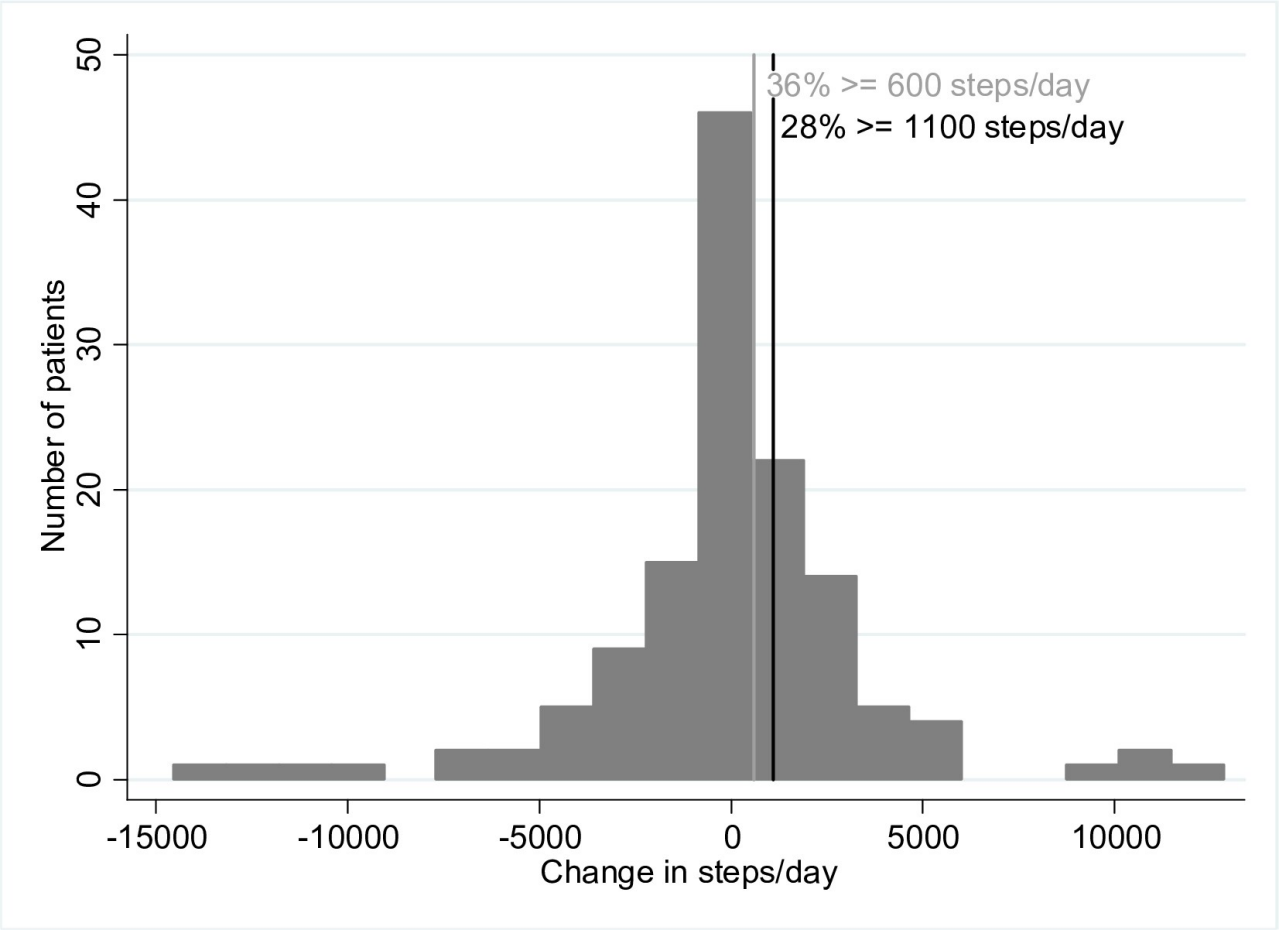
Change in steps/day between baseline and 12-month follow-up after a behavioral physical activity intervention.

Among patients who completed the study, 37 patients (28%) qualified as responders using as threshold 1100 steps/day (Fig 1). The change in steps/day between baseline and 12 months was heterogeneous and ranged between less than minus 10,000 and more than plus 10,000 steps/day following a normal distribution (mean (SD) -67 (3648) steps/day) (Fig 2).

Active working, diagnosis of an endocrino-metabolic disease, unwillingness to follow the physical activity intervention, and FVC at baseline were related to the response to the intervention on bivariable analysis (Table 3).

**Table 3:** Variables related to 12-month response in COPD patients participating in a behavioral physical activity intervention.

	Non-responders (12-month change in steps/day <1100)	Responders (12-month change in steps/day ≥1100)	p-value
	n = 95*	n = 37*	
**Sociodemographic**			
Age (years), m (SD)	69.2 (8.7)	66.0 (9.7)	0.075
Sex: male, n (%)	85 (89)	29 (78)	0.095
Smoking status, current, n (%)	24 (25)	10 (27)	0.835
Socioeconomic status, IIIM-IV-V, n (%)	66 (70)	27 (73)	0.754
**Interpersonal**			
Living with a partner**, n (%)	75 (79)	29 (78)	0.943
Grandparenting, n (%)	38 (40)	9 (24)	0.084
Active workers, n (%)	10 (11)	9 (24)	0.043
**Environmental**			
Urban vulnerability index (from 0 -lowest to 1 –highest), m (SD)	0.60 (0.18)	0.63 (0.15)	0.494
Recruitment season			
Spring, n (%)	24 (25)	9 (24)	0.921
Summer, n (%)	9 (9)	2 (6)	
Fall, n (%)	33 (35)	13 (35)	
Winter, n (%)	29 (31)	13 (35)	
**Clinical**			
FEV_1_ (% pred), m (SD)	54.3 (16.4)	60.1 (17.1)	0.074
FVC (% pred), m (SD)	75.7 (16.3)	82.2 (17.9)	0.048
6MWD (m), m (SD)	493 (97)	512 (90)	0.299
Moderate to very severe dyspnea (mMRC ≥2), n (%)	27 (28)	9 (24)	0.635
Any severe^¶^ COPD exacerbation in previous 12 months, n (%)	9 (10)	1 (3)	0.282
Any severe^¶^ COPD exacerbation during follow-up, n (%)	13 (14)	9 (26)	0.123
BMI (kg/m^2^), m (SD)	28.4 (5.3)	28.4 (4.1)	0.998
FFMI, m (SD)	19.6 (3.3)	19.4 (2.4)	0.690
Neoplasm^†^, n (%)	14 (15)	5 (14)	0.902
Endocrine, nutritional and metabolic diseases^†^, n (%)	58 (61)	31 (86)	0.006
Diabetes mellitus^†^, n (%)	31 (33)	13 (36)	0.707
Cardiovascular disease^†^, n (%)	58 (61)	23 (64)	0.765
Hypertension^†^, n (%)	41 (43)	20 (56)	0.204
Steps/day, m (SD)	8241 (4824)	7625 (3794)	0.487
Time in moderate to vigorous physical activity (>3 METs; h/day), med (P25-P75)	1.7 (1-3-2.4)	1.8 (1.2–2.4)	0.933
Intensity during physical activities (m/s^2^), m (SD)	1.09 (0.31)	1.05 (0.27)	0.431
C-PPAC amount, med (P25-P75)	77 (67–83)	77 (70–91)	0.238
C-PPAC difficulty, med (P25-P75)	86 (75–94)	85 (73–94)	0.908
C-PPAC score, med (P25-P75)	78 (73–86)	83 (68–89)	0.423
**Psychological**			
Anxiety (HAD-A), m (SD)	5.3 (4.2)	5.4 (3.7)	0.861
Depression (HAD-D), m (SD)	3.8 (3.5)	3.6 (3.3)	0.810
Unwillingness to follow the intervention, n (%)	22 (23)	2 (5)	0.022
Stage of change: action, maintaining, finalizing^§^, n (%)	44 (56)	14 (50)	0.559
Self-efficacy^‡^ (0 to 10), med (P25-P75)	8 (7–10)	8 (6–10)	0.755

Data are presented as n (%), mean (SD) or median (P25-P75). FEV_1_: forced expiratory volume in 1 second; FVC: forced vital capacity; 6MWD: 6-min walking distance; mMRC: modified Medical Research Council; BMI: body mass index; FFMI: fat free mass index; MET: metabolic equivalent of task; C-PPAC: Clinical visit—PROactive Physical Activity in COPD; HAD-A: Hospital Anxiety and Depression scale- Anxiety; HAD-D: Hospital Anxiety and Depression scale- Depression.

** Living with a partner vs single, widowed or divorced.

^¶^ A COPD exacerbation was considered severe if the patient required admission to the hospital or the emergency department.

^†^ ICD10 codes: C00 to D48 for Neoplasm; E00 to E90 for Endocrine, nutritional and metabolic diseases; E10 to E14 for Diabetes mellitus; I00 to I99 for Cardiovascular diseases; I10 to I15 for Hypertension.

^§^ Stage of change: action, maintaining, finalizing vs pre-contemplation, contemplation, preparation.

^‡^ Self-efficacy: Sure to go out for a walk every day (0 not sure- 10 completely sure).

* Some variables had missing values. Number of missings for 12-month response: 1 in socioeconomic status, 1 in grandparenting, 1 in urban vulnerability index, 2 for severe COPD exacerbation in previous 12 months, 5 for severe COPD exacerbation during follow-up, 12 for FFMI, 1 for neoplasm, 1 for endocrine, nutritional and metabolic diseases, 1 for diabetes mellitus, 1 for any cardiovascular disease, 1 for hypertension, 35 for C-PPAC, 1 in depression, 26 in stage of change, 8 in self-efficacy.

After mutual adjustment in a multivariable model, being an active worker (3.14 [1.05–9.33], p = 0.040) and having an endocrino-metabolic disease (4.36 [1.49–12.80], p = 0.007) were independently related to a higher probability of response while unwillingness to follow the intervention (0.21 [0.05–0.098], p = 0.047) was related to a lower probability (Table 4).

**Table 4:** Adjusted predictive factors of 12-month response to a behavioral physical activity intervention in 132 COPD patients.

	OR (95% CI)	p-value
Active workers	3.14 (1.05–9.33)	0.040
Endocrine, nutritional and metabolic diseases^†^	4.36 (1.49–12.80)	0.007
Unwillingness to follow the intervention	0.21 (0.05–0.98)	0.047

OR: odds ratio; CI: confidence interval.

^†^ ICD10 codes: E00 to E90 for endocrine, nutritional and metabolic diseases.

### Models fitting and additional analyses

The LASSO approach confirmed that final models of determinants of completion and response (Tables 2 and 4) were not overadjusted. Goodness of fit tests did not reveal any abnormality. Final models indicated good discrimination, as per AUROC of 0.73 for study completion and 0.71 for intervention response, and accuracy with Brier scores lower than 0.2 in both models. All secondary and sensitivity analyses provided very similar results (Tables A-H in S1 File).

## Discussion

This study about the determinants of 12-month completion and response to a behavioral physical activity intervention in COPD patients has found that: (1) being more physically active and living with a partner were positively associated with study completion while living in more vulnerable neighborhoods was negatively associated; (2) active working and endocrino-metabolic comorbidities were positively associated with intervention response while unwillingness to follow the intervention was negatively associated; (3) most clinical and functional characteristics of COPD previously reported to be relevant for physical activity and specifically interventions‘ completion or response were not associated with these outcomes in our sample population; and (4) predictors of study completion were different from predictors of intervention response.

### Determinants of study completion

Our study shows that patients who were more physically active at baseline were more likely to complete the study. We hypothesize that physically active patients already had established a habit around regular physical activity, which acted as a facilitator for participation so that these patients were willing and able to complete the study. Indeed, continuation of an active lifestyle from the past has been reported as a reason to be physically active in an earlier mixed-methods study which combined qualitative and quantitative approaches [32]. Thus, while some intervention studies have excluded more active patients, our results support the inclusion of all COPD patients, even those already considerably active at baseline, in line with the GOLD strategy to promote physical activity for all patients [5].

Living with a partner or in a less vulnerable neighborhood were further predictors of study completion. The role of interpersonal factors such as social support from family, or environmental factors (e.g. ‘*seeing others active‘* or *‘neighborhood walkability‘*), has been recognized within the ecological model of the determinants of physical activity in the general population [14] and is increasingly studied in COPD patients. Being in a supportive relationship was associated with higher levels of physical activity in COPD patients [22,33]. Qualitative studies on barriers to pulmonary rehabilitation have linked living alone to a lack of support, increased challenges to participate, and reduced motivation [34,35]. We anticipate that both living with a partner and living in a less vulnerable neighborhood may facilitate study completion through mechanisms such as social support, behavioral modeling and walkable access to public open spaces [14], thereby lowering mental and physical barriers.

### Determinants of intervention response

Being an active worker was related to a higher likelihood of response to the Urban Training intervention. This finding was surprising since one might expect that working actively might hinder a response to the intervention for time constraints. Lack of time has been acknowledged as a barrier to physical activity in the general [36] and in the COPD population [37] and working is often used as excuse not to exercise. Several hypotheses might explain this unexpected association. First, active workers were younger (mean age 59 *vs* 70 years in our sample, p<0.001), which could have increased their capacities to engage in the intervention. However, in our study age was not related with response (Table 3). Second, patients who kept working despite their COPD, may have had better overall health conditions than those who were retired (which was supported by higher exercise capacity in this group, median 6MWD 540 vs 508, p = 0.139). However, our data does not support a role for exercise capacity in response to the intervention (Table 3). We speculate that active workers may have a more active attitude and higher social support (by work colleagues) to engage in an intervention. They also may have experienced more opportunities to integrate a new physical activity habit into daily life routine and change to a more active life style e.g. switching the commuting mode from private car to walking.

Diagnosis of an endocrino-metabolic comorbidity was also significantly associated with higher likelihood of intervention response, which could seem counterintuitive if patients with concomitant diseases have a poorer health status. In the previous study by Demeyer et al the response to the telecoaching intervention was similar in patients with more than two comorbidities compared to none or one comorbidity [8]. Unfortunately, specific comorbidities were not investigated in that study which precludes direct comparison. Similarly, in the previous study by Moy the number of comorbidities did not predict a change in steps/day at 4 months [12]. Akin to our results, metabolic disease has been reported as an independent predictor for higher response to pulmonary rehabilitation in COPD patients [38]. In our specific sample, endocrino-metabolic comorbidity included mostly a physician diagnosis of diabetes, overweight/obesity and/or hyperlipidemia. However, the small number of cases combined with the high variability in the outcome (response to the intervention) preclude the analysis of the individual effect of each chronic condition. We speculate that the diagnosis of some comorbidities prior to our intervention may have led to the recommendation of increased physical activity in the past. Raised awareness and sensitivity to the topic may in turn have motivated response to the Urban Training intervention.

Spontaneously expressed unwillingness to follow the intervention instructions at baseline was inversely related to the likelihood of response. This is in line with the Urban Training principal paper which observed the intervention to be efficacious to increase physical activity in the *per protocol* population but ineffective in the full population [9]. It also underlines clearly the important role of motivation and a positive mindset towards the physical activity intervention, which have been established previously as important factors for physical activity in qualitative and mixed-methods cross-sectional studies with COPD patients [32,37,39]. Unfortunately, our study did not collect quantitative data on motivation. However, it could be argued that both being an active worker and having a diagnosis of endocrino-metabolic comorbidities essentially also reflect the importance of motivation.

There was a number of variables we had expected to be associated with the intervention response, based on the results of previous studies [8,11,13], on research on physical activity and COPD [2] and on clinical experience. These variables included markers of COPD severity (FEV_1_, COPD exacerbations, dyspnea or exercise capacity), psychological factors (depression, anxiety or self-efficacy), the season of inclusion, or the baseline levels of physical activity. Whether their lack of association with intervention response may be due to the fact that other studies recruited patients in more advanced stages of disease needs to be confirmed in further clinical trials.

### Implications

Our study has implications for future research, clinical management and public health and policies. First, the response to the Urban Training intervention (defined as an increase of ≥1100 steps/day after 12 months, according to previously published MID [28]) has been low with 28% which is in line with the telecoaching intervention which observed a 36% response (defined as an increase of > 1000 steps/day) [8]. This reinforces the need to understand which patients would benefit most from the available resources. Second, we may consider excluding patients who reject participation at baseline to contain health service costs and maximize effectiveness, and consider alternative strategies for these patients. Third, physical activity interventions should neither exclude patients with certain chronic conditions nor actively working patients but rather pay special attention to these individuals since they may be particularly motivated and thus respond better. Fourth, the role of baseline levels of physical activity to include or exclude COPD patients into physical activity interventions should be cautiously considered, since existing literature is still unclear and no patient should be left without an intervention potentially efficacious for him/her. Finally, we suggest that screening for interpersonal and environmental factors may help to decide which patients are more likely to succeed in long-term physical activity interventions.

### Strengths and limitations

A major strength of our study lies within the fact that the Urban Training intervention was administered to COPD patients from hospital and primary care settings. The findings therefore reflect more closely than previous studies (based on hospitals or rehabilitation settings only) what can be expected when deploying a physical activity intervention at the population level. Moreover, the study design allowed us to assess the determinants of a long-term effect. These are more likely to be the determinants of a real behavior change as compared to the short-term effects shown in literature. Finally, the broad number of variables included in our study reflect well the various domains of the determinants of physical activity within the ecological framework [14].

As potential limitation we have to acknowledge the small sample size that did not allow testing the potential association of some rare factors (e.g. less frequent comorbidities) with completion or response. Although we included a broad range of variables, some variables related to physical activity limitation in COPD patients such as reduced muscle strength or impaired lung volumes were missing. Finally, our study population reflects the behavior of a population from a specific geographic area (i.e. ‘*elderly inhabitants of Mediterranean cities’* [9]) and included a large proportion of patients with relatively mild-to-moderate stages of disease. Thus, further research is needed to identify determinants of completion and response in patients from other regions or medical settings, as is usually done prior to deployment of any intervention.

## Conclusions

Among a broad range of potential predictors of 12-month completion or response to a behavioral physical activity intervention, this study found that completion was generally determined by previous physical activity habits as well as interpersonal and environmental physical activity facilitators while response was related to diverse factors thought to modify the individual motivation to change to an active lifestyle. These results support a look beyond the traditional clinical and functional variables and the consideration of psychological, interpersonal and environmental factors related to habits and motivation to optimize the outcome of physical activity interventions in COPD.

## Supporting information

S1 File Supporting information. (PDF)
