# Supplementary material for: Determinants of study completion and response to a 12-month behavioral physical activity intervention in chronic obstructive pulmonary disease: A cohort study
Source: PLoS One. 2019 May 20;14(5):e0217157. doi: 10.1371/journal.pone.0217157 (PMC6527234; doi:10.1371/journal.pone.0217157)
Supplement: S1 File — (PDF) [file pone.0217157.s001.pdf]

S1 File. Supporting Information.

Determinants of study completion and response to a 12-month behavioral physical activity intervention in chronic obstructive pulmonary disease: A cohort study

Maria Koreny, Heleen Demeyer, Ane Arbillaga-Etxarri, Elena Gimeno-Santos, Anael Barberan-Garcia, Marta Benet, Eva Balcells, Eulàlia Borrell, Alicia Marin, Diego A. Rodríguez Chiaradía, Pere Vall-Casas, Jordi Vilaró, Robert Rodríguez-Roisin, Judith Garcia-Aymerich

Tables:

Table A. Variables related to 12-month response to a behavioral physical activity intervention using 600 steps/day as cut-off for response.

Table B. Adjusted predictive factors of 12-month response to a behavioral physical activity intervention using 600 steps/day as cut-off for response.

Table C. Adjusted predictive factors of 12-month response to a behavioral physical activity intervention including exacerbations during follow-up as a covariate.

Table D. Adjusted predictive factors of 12-month response to a behavioral physical activity intervention using change in moderate to vigorous physical activity to define response#.

Table E. Variables related to 12-month completion in COPD patients participating in a behavioral physical activity intervention using multiple imputation.

Table F. Adjusted predictive factors of 12-month completion of a behavioral physical activity intervention in 202 COPD patients using multiple imputation.

Table G. Variables related to 12-month response in COPD patients participating in a behavioral physical activity intervention using multiple imputation.

Table H. Adjusted predictive factors of 12-month response to a behavioral physical activity intervention in 132 COPD patients using multiple imputation.

Table A. Variables related to 12-month response to a behavioral physical activity intervention <u>using 600 steps/day as cut-off for response</u>.

	Non-responders (12-month change in steps/day <600)	Responders (12-month change in steps/day ≥600)	p-value
	n=85*	n=47*	
**Sociodemographic**			
Age (years), m (SD)	69.2 (8.3)	66.7 (10.2)	0.138
Sex: male, n (%)	76 (89)	38 (81)	0.170
Smoking status, current, n (%)	21 (25)	13 (28)	0.710
Socioeconomic status, IIIM-IV-V, n (%)	57 (68)	36 (77)	0.290
**Interpersonal**			
Living with a partner**, n (%)	66 (78)	38 (81)	0.666
Grandparenting, n (%)	36 (43)	11 (23)	0.026
Active workers, n (%)	9 (11)	10 (21)	0.094
**Environmental**			
Urban vulnerability index (from 0 -lowest to 1 –highest), m (SD)	0.61 (0.18)	0.61 (0.15)	0.853
Recruitment season			
Spring, n (%)	23 (27)	10 (21)	0.773
Summer, n (%)	8 (9)	3 (7)	
Fall, n (%)	29 (34)	17 (36)	
Winter, n (%)	25 (30)	17 (36)	
**Clinical**			
FEV_1 (% pred), m (SD)	54.4 (16.7)	58.7 (16.6)	0.152
FVC (% pred), m (SD)	76.1 (16.6)	80.1 (17.3)	0.195
6MWD (m), m (SD)	491 (100)	512 (84)	0.240
Moderate to very severe dyspnea (mMRC ≥2), n (%)	25 (29)	11 (23)	0.458
Any severe[¶] COPD exacerbation in previous 12 months, n (%)	9 (11)	1 (2)	0.097
Any severe[¶] COPD exacerbation during follow-up, n (%)	12 (15)	10 (22)	0.330
BMI (kg/m^2), m (SD)	28.5 (5.4)	28.1 (4.1)	0.669
FFMI, m (SD)	19.7 (3.2)	19.3 (2.8)	0.417
Neoplasm[†], n (%)	12 (14)	7 (15)	0.865
Endocrine, nutritional and metabolic diseases[†], n (%)	52 (61)	37 (80)	0.024
Diabetes mellitus[†], n (%)	29 (34)	15 (33)	0.861
Any cardiovascular disease[†], n (%)	52 (61)	29 (63)	0.834
Hypertension[†], n (%)	37 (44)	24 (52)	0.344
Steps/day, m (SD)	8397 (4949)	7474 (3709)	0.267
Time in moderate to vigorous physical activity (>3 METs; h/day), med (P25-P75)	1.7 (1.3-2.4)	1.8 (1.2-2.4)	0.938
Intensity during physical activities (m/s^2), m (SD)	1.06 (0.27)	1.06 (0.30)	0.939
C-PPAC amount, med (P25-P75)	77 (63-83)	77 (72-91)	0.194
C-PPAC difficulty, med (P25-P75)	86 (77-94)	83 (73-94)	0.382
C-PPAC score, med (P25-P75)	78 (72-86)	78 (69-89)	0.617
**Psychological**			
Anxiety (HAD-A), m (SD)	5.3 (4.2)	5.4 (3.8)	0.917
Depression (HAD-D), m (SD)	3.8 (3.5)	3.6 (3.2)	0.718
Unwillingness to follow the intervention, n (%)	21 (25)	3 (6)	0.009
Stage of change: action, maintaining, finalizing[§], n (%)	40 (57)	18 (50)	0.484
Self-efficacy[‡] (0 to 10), med (P25-P75)	8 (6-10)	9 (7-10)	0.145

Data are presented as n (%), mean (SD) or median (P25-P75). FEV_1: forced expiratory volume in 1 second; FVC: forced vital capacity; 6MWD: 6-min walking distance; mMRC: modified Medical Research Council; BMI: body mass index; FFMI: fat free mass index; MET: metabolic equivalent of task; C-PPAC: Clinical visit - PROactive Physical Activity in COPD; HAD-A: Hospital Anxiety and Depression scale- Anxiety; HAD-D: Hospital Anxiety and Depression scale- Depression.

** Living with a partner vs single, widowed or divorced.

¶ A COPD exacerbation was considered severe if the patient required admission to the hospital or the emergency department.

† ICD10 codes: C00 to D48 for Neoplasm; E00 to E90 for Endocrine, nutritional and metabolic diseases; E10 to E14 for Diabetes mellitus; I00 to I99 for Cardiovascular diseases; I10 to I15 for Hypertension.

§ Stage of change: action, maintaining, finalizing vs pre-contemplation, contemplation, preparation.

‡ Self-efficacy: Sure to go out for a walk every day (0 not sure- 10 completely sure).

* Some variables had missing values. Number of missings for 12-month response: 1 in socioeconomic status, 1 in grandparenting, 1 in urban vulnerability index, 2 for severe COPD exacerbation in previous 12 months, 5 for severe COPD exacerbation during follow-up, 12 for FFMI, 1 for neoplasm, 1 for endocrine, nutritional and metabolic diseases, 1 for diabetes mellitus, 1 for any cardiovascular disease, 1 for hypertension, 35 for C-PPAC, 1 in depression, 26 in stage of change, 8 in self-efficacy.

Table B. Adjusted predictive factors of 12-month response to a behavioral physical activity intervention using 600 steps/day as cut-off for response.

	OR (95% CI)	p-value
Endocrine, nutritional and metabolic diseases†, n (%)	2.78 (1.17-6.61)	0.021
Active workers, n (%)	2.60 (0.94-7.20)	0.067

OR: odds ratio; CI: confidence interval.

† ICD10 codes: E00 to E90 for endocrine, nutritional and metabolic diseases.

Table C. Adjusted predictive factors of 12-month response to a behavioral physical activity intervention including exacerbations during follow-up as a covariate.

	Main Model OR (95% CI)	p-value	Model with exacerbations OR (95% CI)	p-value
Active workers	3.14 (1.05-9.33)	0.040	2.86 (0.93-8.77)	0.066
Endocrine, nutritional and metabolic diseases†	4.36 (1.49-12.80)	0.007	4.45 (1.49-13-29)	0.008
Unwillingness to follow the intervention	0.21 (0.05-0.98)	0.047	0.18 (0.04-0.91)	0.038
Any severe¶ COPD exacerbation during follow-up	-	-	2.65 (0.92-7.64)	0.071

OR: odds ratio; CI: confidence interval.

† ICD10 codes: E00 to E90 for endocrine, nutritional and metabolic diseases.

¶ A COPD exacerbation was considered severe if the patient required admission to the hospital or the emergency department.

Table D. Adjusted predictive factors of 12-month response to a behavioral physical activity intervention using change in moderate to vigorous physical activity to define response#.

	OR (95% CI)	p-value
Active workers	1.45 (0.52-4.04)	0.474
Endocrine, nutritional and metabolic diseases†	2.31 (1.07-5.01)	0.034
Unwillingness to follow the intervention	0.36 (0.14-0.97)	0.042

OR: odds ratio; CI: confidence interval.

# We used the median change of time in moderate to vigorous physical activity (between visit 2 and visit 4) as cut-off to define response to a behavioural physical activity intervention.

† ICD10 codes: E00 to E90 for endocrine, nutritional and metabolic diseases.

Table E. Variables related to 12-month completion in COPD patients participating in a behavioral physical activity intervention using multiple imputation.

	All patients	Lost to follow-up	Completers	p-value
	n=202	n=70	n=132	
**Sociodemographic**				
Age (years), m (SD)	68.8 (9.2)	69.9 (9.3)	68.3 (9.1)	0.229
Sex: male, n (%)	170 (84)	56 (80)	114 (86)	0.241
Smoking status, current, n (%)	56 (28)	22 (31)	34 (26)	0.392
Socioeconomic status, IIIM-IV-V, n (%)	144 (71)	50 (71)	94 (71)	0.965
**Interpersonal**				
Living with a partner**, n (%)	145 (72)	41 (59)	104 (79)	0.004
Grandparenting, n (%)	69 (34)	22 (31)	47 (36)	0.482
Active workers, n (%)	28 (14)	9 (13)	19 (14)	0.764
**Environmental**				
Urban vulnerability index (from 0 -lowest to 1 –highest), m (SD)	0.64 (0.17)	0.69 (0.16)	0.61 (0.17)	0.003
Recruitment season				
Spring, n (%)	46 (23)	13 (19)	33 (25)	0.316
Summer, n (%)	22 (11)	11 (16)	11 (8)	
Fall, n (%)	73 (36)	27 (39)	46 (35)	
Winter, n (%)	61 (30)	19 (27)	42 (32)	
**Clinical**				
FEV_1 (% pred), m (SD)	56.4 (17.1)	57.2 (17.9)	55.9 (16.7)	0.614
FVC (% pred), m (SD)	77.3 (16.8)	76.9 (16.8)	77.5 (16.9)	0.816
6MWD (m), m (SD)	487 (98)	464 (102)	498 (95)	0.021
Moderate to very severe dyspnea (mMRC ≥2), n (%)	58 (29)	22 (31)	36 (27)	0.535
Any severe[¶] COPD exacerbation in previous 12 months, n (%)	19 (10)	9 (13)	10 (8)	0.301
Any severe[¶] COPD exacerbation during follow-up, n (%)	22 (17)	-	22 (17)	-
BMI (kg/m^2), m (SD)	28.5 (5.0)	28.6 (5.1)	28.4 (4.9)	0.811
FFMI, m (SD)	19.6 (3.2)	19.4 (3.5)	19.6 (3.1)	0.682
Neoplasm[†], n (%)	25 (12)	5 (8)	20 (15)	0.169
Endocrine, nutritional and metabolic diseases[†], n (%)	134 (66)	44 (63)	90 (68)	0.522
Diabetes mellitus[†], n (%)	63 (31)	18 (26)	44 (34)	0.308
Cardiovascular disease[†], n (%)	127 (63)	45 (65)	81 (62)	0.664
Hypertension[†], n (%)	96 (48)	35 (50)	62 (47)	0.702
Steps/day, m (SD)	7488 (4234)	6395 (3315)	8069 (4554)	0.009
Time in moderate to vigorous physical activity (>3 METs; h/day), med (P25-P75)	1.7 (1.2-2.2)	1.6 (1.1-2.0)	1.7 (1.3-2.4)	0.025
Intensity during physical activities (m/s^2), m (SD)	1.03 (0.27)	0.97 (0.23)	1.06 (0.28)	0.022
C-PPAC amount, med (P25-P75)	77 (67-83)	72 (67-83)	77 (67-83)	0.406
C-PPAC difficulty, med (P25-P75)	84 (73-94)	84 (71-94)	85 (74-94)	0.924
C-PPAC score, med (P25-P75)	79 (70-86)	79 (69-84)	79 (72-87)	0.456
**Psychological**				
Anxiety (HAD-A), m (SD)	5.4 (4.2)	5.6 (4.6)	5.3 (4.0)	0.636
Depression (HAD-D), m (SD)	3.6 (3.7)	3.3 (4.1)	3.8 (3.4)	0.435
Unwillingness to follow the intervention, n (%)	26 (13)	2 (3)	24 (18)	0.007
Stage of change: action, maintaining, finalizing[§], n (%)	106 (53)	32 (45)	75 (57)	0.136
Self-efficacy[‡] (0 to 10), med (P25-P75)	8 (7-10)	8 (6-10)	8 (7-10)	0.444

Data are presented as n (%), mean (SD) or median (P25-P75). FEV_1: forced expiratory volume in 1 second; FVC: forced vital capacity; 6MWD: 6-min walking distance; mMRC: modified Medical Research Council; BMI: body mass index; FFMI: fat free mass index; MET: metabolic equivalent of task; C-PPAC: Clinical visit - PROactive Physical Activity in COPD; HAD-A: Hospital Anxiety and Depression scale- Anxiety; HAD-D: Hospital Anxiety and Depression scale- Depression.

** Living with a partner vs single, widowed or divorced.

¶ A COPD exacerbation was considered severe if the patient required admission to the hospital or the emergency department.

† ICD10 codes: C00 to D48 for Neoplasm; E00 to E90 for Endocrine, nutritional and metabolic diseases; E10 to E14 for Diabetes mellitus; I00 to I99 for Cardiovascular diseases; I10 to I15 for Hypertension.

§ Stage of change: action, maintaining, finalizing vs pre-contemplation, contemplation, preparation.

‡ Self-efficacy: Sure to go out for a walk every day (0 not sure- 10 completely sure).

Table F. Adjusted predictive factors of 12-month completion of a behavioral physical activity intervention in 202 COPD patients using multiple imputation.

	OR (95% CI)	p-value
Steps/day (per increase of 1000 steps)	1.11 (1.03-1.21)	0.010
Living with a partner (vs single/ widowed/ divorced)	2.67 (1.35-5.23)	0.005
Urban vulnerability index (per increase of 0.1 units)	0.71 (0.58-0.88)	0.001

OR: odds ratio; CI: confidence interval.

Table G. Variables related to 12-month response in COPD patients participating in a behavioral physical activity intervention using multiple imputation.

	Non-responders (12-month change in steps/day <1100)	Responders (12-month change in steps/day ≥1100)	p-value
	n=95	n=37	
**Sociodemographic**			
Age (years), m (SD)	69.2 (8.7)	66.0 (9.7)	0.077
Sex: male, n (%)	85 (89)	29 (78)	0.102
Smoking status, current, n (%)	24 (25)	10 (27)	0.835
Socioeconomic status, IIIM-IV-V, n (%)	67 (70)	27 (73)	0.772
**Interpersonal**			
Living with a partner**, n (%)	75 (79)	29 (78)	0.943
Grandparenting, n (%)	38 (40)	9 (24)	0.090
Active workers, n (%)	10 (11)	9 (24)	0.048
**Environmental**			
Urban vulnerability index (from 0 -lowest to 1 –highest), m (SD)	0.60 (0.18)	0.63 (0.15)	0.515
Recruitment season			
Spring, n (%)	24 (25)	9 (24)	0.873
Summer, n (%)	9 (9)	2 (5)	
Fall, n (%)	33 (35)	13 (35)	
Winter, n (%)	29 (31)	13 (35)	
**Clinical**			
FEV_1 (% pred), m (SD)	54.3 (16.4)	60.1 (17.1)	0.077
FVC (% pred), m (SD)	75.7 (16.3)	82.2 (17.9)	0.052
6MWD (m), m (SD)	493 (97)	512 (90)	0.299
Moderate to very severe dyspnea (mMRC ≥2), n (%)	27 (28)	9 (24)	0.635
Any severe¶ COPD exacerbation in previous 12 months, n (%)	9 (10)	1 (3)	0.210
Any severe¶ COPD exacerbation during follow-up, n (%)	13 (14)	9 (25)	0.149
BMI (kg/m^2), m (SD)	28.4 (5.3)	28.4 (4.1)	0.998
FFMI, m (SD)	19.7 (3.3)	19.5 (2.6)	0.856
Neoplasm†, n (%)	14 (15)	6 (15)	0.929
Endocrine, nutritional and metabolic diseases†, n (%)	58 (61)	32 (86)	0.011
Diabetes mellitus†, n (%)	31 (33)	13 (36)	0.731
Cardiovascular disease†, n (%)	58 (61)	23 (63)	0.806
Hypertension†, n (%)	41 (43)	21 (56)	0.207
Steps/day, m (SD)	8241 (4824)	7625 (3794)	0.484
Time in moderate to vigorous physical activity (>3 METs; h/day), med (P25-P75)	1.7 (1.3-2.4)	1.8 (1.2-2.4)	0.805
Intensity during physical activities (m/s^2), m (SD)	1.05 (0.27)	1.09 (0.31)	0.430
C-PPAC amount, med (P25-P75)	77 (65-83)	77 (68-85)	0.347
C-PPAC difficulty, med (P25-P75)	85 (74-94)	84 (73-94)	0.620
C-PPAC score, med (P25-P75)	79 (72-86)	81 (69-89)	0.749
**Psychological**			
Anxiety (HAD-A), m (SD)	5.3 (4.2)	5.4 (3.7)	0.859
Depression (HAD-D), m (SD)	3.8 (3.5)	3.7 (3.4)	0.859

Unwillingness to follow the intervention, n (%)	22 (23)	2 (5)	0.030
Stage of change: action, maintaining, finalizing§, n (%)	54 (57)	20 (55)	0.836
Self-efficacy‡ (0 to 10), med (P25-P75)	8 (7-10)	8 (6-10)	0.736

Data are presented as n (%), mean (SD) or median (P25-P75). FEV_1: forced expiratory volume in 1 second; FVC: forced vital capacity; 6MWD: 6-min walking distance; mMRC: modified Medical Research Council; BMI: body mass index; FFMI: fat free mass index; MET: metabolic equivalent of task; C-PPAC: Clinical visit - PROactive Physical Activity in COPD; HAD-A: Hospital Anxiety and Depression scale- Anxiety; HAD-D: Hospital Anxiety and Depression scale- Depression.

** Living with a partner vs single, widowed or divorced.

¶ A COPD exacerbation was considered severe if the patient required admission to the hospital or the emergency department.

† ICD10 codes: C00 to D48 for Neoplasm; E00 to E90 for Endocrine, nutritional and metabolic diseases; E10 to E14 for Diabetes mellitus; I00 to I99 for Cardiovascular diseases; I10 to I15 for Hypertension.

§ Stage of change: action, maintaining, finalizing vs pre-contemplation, contemplation, preparation.

‡ Self-efficacy: Sure to go out for a walk every day (0 not sure- 10 completely sure).

Table H. Adjusted predictive factors of 12-month response to a behavioral physical activity intervention in 132 COPD patients using multiple imputation.

	OR (95% CI)	p-value
Active workers	3.00 (1.01-8.90)	0.048
Endocrine, nutritional and metabolic diseases†	4.19 (1.42-12.32)	0.009
Unwillingness to follow the intervention	0.20 (0.04-0.94)	0.042

OR: odds ratio; CI: confidence interval.

† ICD10 codes: E00 to E90 for endocrine, nutritional and metabolic diseases.
